# Novel Approaches in Diagnosing the Role of Inflammation in the Onset Cardiovascular Disorders

**DOI:** 10.1155/2018/8234063

**Published:** 2018-10-18

**Authors:** Adrian Doroszko, Piotr Dobrowolski, Aneta Radziwon-Balicka, Robert Skomro

**Affiliations:** ^1^Department of Internal Medicine, Occupational Diseases and Hypertension, Wroclaw Medical University, Wroclaw, Poland; ^2^Department of Congenital Heart Diseases, Institute of Cardiology, Warsaw, Poland; ^3^Department of Clinical Experimental Research, Glostrup Research Institute, Rigshospitalet, Glostrup, Denmark; ^4^Division of Respirology, Critical Care and Sleep Medicine, Department of Medicine, University of Saskatchewan, Saskatoon, Canada

Cardiovascular disease remains among the major healthcare problems of the world population, and understanding its determinants is pivotal for designing novel effective interventions. Advances in molecular medicine have enabled us to identify the critical pathways involved in cell survival or death in the myocardium, endothelial cells, vascular wall, and brain. What is more, some crucial regulators of these pathways have been newly identified, resulting in the development of novel strategies for treatment of coronary artery disease, hypertension, and congestive heart failure. There are a number of chemical mediators and pathways involved in the inflammatory onset of cardiovascular disorders. Thus, from a therapeutic point of view, it would be most important to focus on the active mediators of inflammation and apoptosis and to manipulate these in order to improve cell function and survival. The cellular mechanisms involved in the pathogenesis of cardiovascular inflammatory injury are complex and involve the interactions of various cells, including coronary endothelial cells, circulating blood cells, and cardiac myocytes. The intracellular signalling pathways that mediate stress responses and determine cell death or survival have not been fully investigated. Protein kinase activation potentially regulates the onset of injury. A substantial amount of basic research has defined many of the details regarding the role of inflammation in the kinase pathway organization and activation; nevertheless, the role of individual signalling pathways in pathogenesis of various forms of cardiovascular disease is still investigated.

This special issue is aiming at stimulating the continuing effort to understand the molecular mechanisms underlying cardiovascular damage mediated by inflammation.

Selenoprotein S (SelS) has been identified in endothelial cells and is associated with inflammation. S. Cui et al. demonstrated that the upregulation of SelS enhances the levels of nitric oxide and endothelial nitric oxide synthase in tumour necrosis factor-*α*-treated human umbilical vein endothelial cells. The authors postulate that SelS protects endothelial cells against TNF-*α*-induced dysfunction by inhibiting the activation of p38 MAPK and NF-*κ*B pathways and implicate it as a possible modulator of vascular inflammatory diseases.

Oxysterols may affect cholesterol metabolism, membrane fluidity regulation, and intracellular signalling pathways. They are implicated in a pathophysiology of type 2 diabetes mellitus and neurodegenerative disorder as well as in malignancies. Some studies postulate that oxysterols may play a role in atherogenesis. T. Wielkoszyński et al., in their experimental study, have shown that the oxidized cholesterol metabolites are postulated to exert atherogenic effect and thus adversely affect vascular endothelium. The authors demonstrate that oxidized cholesterol derivatives exert cytotoxic effect on vascular endothelial cells, causing endothelial dysfunction, the severity of which depends on the duration of exposure. What is more, combined administration of oxysterols and cholesterol was shown to increase their angiotoxic effect.

Despite advances in critical care treatment and increased understanding of the sepsis pathophysiology, the mortality rate of affected patients remains high, ca. 50% even in developed countries, and is mostly attributed to cardiovascular dysfunction. Endothelial dysfunction (ED) occurring with an overproduction of endothelium-derived contracting factor (EDCF) plays a key role in the pathogenesis of cardiovascular disease. Overproduction of nitric oxide and other molecules of vasodilatory action may result in uncontrolled vasodilation leading in turn to the development of hemodynamic shock. W. Han et al., in an interesting paper, found that inhibition of the mTOR pathway plays a cardioprotective role in a sepsis-induced myocardial dysfunction and that this effect may be mediated by acceleration of autophagy. The mTOR is a sensor of energetic status and induces autophagy upon energy depletion, which is a major cause of myocardial dysfunction during sepsis. Hence, the mTOR pathway seems to play an important role in myocardial dysfunction induced by sepsis. The authors found that the mTOR pathway was inhibited and rapamycin significantly alleviated cardiac dysfunction and improved myocardial anoxia in septic cardiomyopathy.

A. Stanek et al. in their study investigate the impact of whole-body cryotherapy on cardiovascular risk factors in patients with ankylosing spondylitis, which—according to the results presented—might be a useful additional method preventing development of atherosclerosis. The authors postulate that the whole-body cryotherapy with subsequent kinesiotherapy may facilitate the decrease in oxidative stress, lipid profile, atherosclerosis plaque, and its instability, as well as inflammatory parameters.

Peripheral artery disease (PAD) affects ca. 25% of population over 60 years old. Inflammation and mitochondrial dysfunction may predispose to PAD, which is associated with other highly prevalent disorders, including diabetes, dyslipidaemia, and hypertension. In a research study by A. Hernández-Aguilera, using metabolomic approach, the authors have demonstrated a relevant correlation between plasma concentrations of energy-balance-associated metabolites, oxidative stress, and inflammation in subjects with peripheral artery disease. The authors postulate that (iso)citrate and glutamate could constitute novel biomarkers for discriminating PAD patients without symptomatic disease.

In summary, the papers presented in this special issue demonstrate the recent developments in understanding the role of inflammation in the onset of cardiovascular disease. We believe that some of the presented studies will provide new evidence, which could lead to the discovery of potential biomarkers and drug targets for the development of novel therapeutic approaches for combating cardiovascular disease in the future.

